# The Hospital. Nursing Section

**Published:** 1903-03-14

**Authors:** 


					The Hospital.
"Rurelno Section, X
Contributions for this Section of "Thb Hospital" should be addressed to the Editor, "Thh Hospital"
Nursing Section, 28 & 29 Southampton Street, Strand, London, W.O.
No. 859.?Vol. XXXIII. SATURDAY, MARCH 14, 1903.
"Motes on iRewa from tbe IRurslng Morlb.
the army nursing question in parliament.
The Secretary of State for War in his speech
"?n the Army Estimates on Tuesday, having re-
ferred to the re-organisation of the Army Medical
department, told the House of Commons that
the Army Nursing Service under the immediate
presidency of her Majesty the Queen?who had
herself a great practical knowledge of nursing?had
been placed on a new footing. "The number of
curses had been largely increased, a considerable
sum was asked for in the Estimates for this service,
and for the first time a proportion of the most highly-
paid and highly-trained male members of the Army
-Medical Corps would be trained as nurses, and would
Qot be put to other departments."
RESIGNATION OF A HOSPITAL MATRON.
Sir Charles Turner, chairman of the Royal
Westminster Ophthalmic Hospital, announced at
^he annual meeting of Governors last week that
news had been received by the committee which
-greatly distressed them. The excellent matron,
Miss Milburn, found herself unable, through fatigue,
continue her duties, and had sent in her [resigna-
tion. The committee greatly regretted her depar-
ture, and trusted that after a period of rest she
would find a post where she might, with renewed
strength, do as much as she had done at the Royal
Westminster Ophthalmic Hospital. Miss Milburn
has occupied her present post for six and a half
years. She was trained at the Royal Infirmary,
Newcastle, and has since been staff nurse at the
Marylebone Infirmary, besides spending a year in
private nursing. Before her appointment to the
Westminster Ophthalmic Hospital, Miss Milburn
spent seven years at the Ophthalmic School, Hanwell.
THE NURSES' HOME AT THE LONDON HOSPITAL.
It was announced by Mr. Sydney Holland, at a
General Court of Governors of the London Hospital
held last week, that orders had been given to proceed
with the drawings and plans for the new Nurses'
Home. The new building will be on the land adjoin-
ing the site intended for the new laundry, and it is
hoped that the Stepney Borough Council will consent
to the erection of a connecting bridge between the
old home and the new one. There are now 497 sisters
and nurses on the staff, many of whom are most
inconveniently lodged in small houses in the neigh-
bourhood of the hospital. The cost of the new home
is estimated at ^45,000.
THE NEW MATRON OF THE CITY OF LONDON
HOSPITAL FOR DISEASES OF THE CHEST.
The successor of Miss Beatrice Jones at the City
of London Hospital for Diseases of the Chest is Miss
Clara E. Trafford, who having been for a time
assistant matron, has acted as matron since the
appointment of Miss Jones to the Herbert Hospital.
There were 55 candidates for the position of matron
and Miss Trafford was unanimously elected by the
committee of management, who must therefore have
been most favourably impressed with the manner in
which she discharged her duties before her election
as head of the nursing staff.
THE SECRETARYSHIP OF THE ROYAL BRITISH
NURSES' ASSOCIATION.
Miss G. A. Leigh has resigned the post of
Secretary of the Royal British Nurses' Association.
The Chairman announced her intention at a meeting
of the Executive Committee, and said that for nearly
six years Miss Leigh had filled the post in an
Maech 14, 1903. THE HOSPITAL. Nursing Section. 325
admirable manner. We learn that the sole reason
for her resignation is that she finds it impossible to
continue to devote the whole of her time and energies
to the work as she has done hitherto ; and that her
interest in the Association is unabated.
SHOREDITCH AND BETHNAL GREEN NURSING
ASSOCIATION.
The annual meeting of the Shoreditch and Bethnal
Green District Nursing Association will be held on
Thursday, March 19, at 3.30, at Londonderry
House, Lady Londonderry presiding. This organ-
isation, which was formerly known as the Haggerston
and Hoxton District Nursing Association, has now
been established thirteen years ; the nurses' home
ls in a square off the Hackney Road in the heart of
the district which the nurses serve, and the staff
consists of the superintendent, three fully-trained
nurses, and seven probationers, who, having received
their hospital training, are sent by the Queen
Victoria Jubilee Institute for six months' training in
district nursing. The services of the nurse are given
tree to all who need them without distinction as to
the religious opinions of her patients ; and as an
illustration of the manner in which they are appreci-
ated, it is mentioned in the report that one of the
Poorest of the patients, in purchasing a sixpenny
ticket for the annual concert?which yielded ?83,
the highest sum reached?explained how she had
collected it from various services, saying, as she did
So> " My husband says we must all rally round the
nurses and support their concert."
CONVERTING A CO-OPERATION INTO A CHARITY.
-Tt appears that even the committee of the Glasgow
^?d West of Scotland Co-operation of Trained
-Nurses are not at all confident that the new con-
stitution will make for prosperity. The constitution
gives them the power "to solicit and collect sub-
scriptions and bequests." This, too, is in spite of
the statement of Lord Provost Chisholm, who at the
annual meeting in November, 1901, said : "The
institution does not appeal for subscriptions. The
nurses themselves by their quarterly and other pay-
ments make the institution not only entirely self-
supporting, but yield such a credit balance that the
heritable property account is rapidly being written
down. I have often had to appeal to the citizens of
Glasgow for subscriptions to support other institu-
tions, but what I have to do on this occasion is to
call attention to the existence of an institution
^hich supports itself." The question should now be
raised why the committee have converted a self-
supporting into a charitable institution, and thus
deprived about 170 nurses, who last year earned
upwards of .?9,000, of the spirit of independence
upon which they had a right to pride themselves.
GUARDIANS AND WORKHOUSE MATRONS.
The sharp difference of opinion which has been
Provoked by that portion of the report of the
departmental committee of the Local Government
-Board on Workhouse Nursing is illustrated by the
receptions given to it by the York and the
Prestwich Guardians. In the judgment of the York
Guardians the matron of the workhouse should be
made co-equal with the master, " as far as her work
as a woman is concerned," and they accordingly
regret her suggested exclusion from the sick wards.
The Prestwich Guardians, on the other hand, have
decided to make an application to the Local Govern-
ment Board for an order to give the superintendent
nurse at the workhouse full control over the infirmary
nurses on the lines indicated in the report of the
departmental committee. We are glad to see that
the York Guardians object to the proposal to create
a new class of " Qualified Nurses " with only one year
of training in a minor training school. For 11 they
consider that probationers, who have served for one
year in a minor training school to the satisfaction of
the medical officer and guardians, should be com-
pelled to complete a further period of two years in a
major training school."
PROGRESS AT WEST BROMWICH.
The reduction of the adverse balance of the
West Bromwich District Nursing Association from
?106 17s. 2d. at the end of 1901, to ?26 lis. 6d. at
the end of 1902 is largely due to one of the dramatic
performances which a Cambridge clergyman so
severely condemns. But there has also been an
increase in the subscriptions of nearly ?100, and
though yet another ?100 per annum is wanted to
place the association on a sound financial basis, the
report for the last twelve months is distinctly
encouraging. The work of the nurses in West
Bromwich was more than sufficiently arduous. The
number of visits made was 15,407, or an average of
1,283 per month. A staff of four nurses, with
partial help from a fifth, is not large enough, and
the superintendent rightly urges the importance o?
adding to it, as soon as the income will justify
further expenditure. As may be supposed, the
Akrill Home, opened by Lord Dartmouth last May,
is greatly appreciated by the nurses ; and the steady
work done by the Ladies' Committee" contributes
substantially to the usefulness of the undertaking.
ONE MORE ILLUSION DISPELLED.
The ignorance of some persons with respect to the
work of a trained nurse dies hard. At the annual
meeting of the Doncaster District Nursing Associa-
tion, which has only been in existence for five years,
Dr. Corbett stated that when the nurses first came
to the town, " one of those dear old ladies who called
themselves nurses said, 1 Whatever's the use of bring-
ing these women in the town to lay people out.'
However, since then the " dear old ladies" have
learned to grasp the fact that the avocation of the
nurses is not limited to laying people out. Last
year, for example, they nursed 245 cases, of which
30 were operation cases, and paid 6,883 visits, an
increase on 1901 of 96 cases and 2,844 visits. The
financial position is good, inasmuch as there is a
small balance in hand, but one of the committee
reminded the members that extraneous help had
been given.
MATERNITY HELP FOR THE POOR.
There was a conference last week at the Grosvenor
Crescent Club, at which certain proposals for the
supply of midwives to the rural and provincial poo^
in accordance with the Act of 1902, were discussed.
No fewer than 26 councils were represented on the
326 Nursing Section. THE HOSPITAL. March 14, 1903.
occasion. Speeches were delivered by the Chairman
(Sir Michael Foster, K.C.B.), Mrs. Hey wood
Johnstone, Dr. Boxall and Mrs. Rickman, who
said that there was not sufficient inducement for
a carefully-trained midwife or nurse to take up
a position in a small district. Mrs. Rickman
suggested that the midwife should be selected from
villages and instructed, and should be allowed at
the same time to follow any other occupation in the
place not detrimental to her calling as nurse or mid-
wife. Subsequently, on the motion of Mr. Heywood
Johnstone, the meeting decided to form an association
to carry into operation plans for a supply of mid-
wives on these lines.
REBELLION AT BARNSLEY.
An incident is reported from Barnsley which,
happily, is very unusual. The Board of Guardians,
owing to an outbreak of small-pox, decided that it
was necessary not to allow the members of the nursing
staff at the workhouse infirmary to leave the institu-
tion. A nurse whose application to go out was
refused tendered her resignation, but instead of
waiting until it took effect she one night last week
left the infirmary by climbing over a garden gate
10 feet high, and did not return. In these circum-
stances the guardians have declined to give her a
testimonial. They are perfectly right. A nurse
who cannot submit to discipline has entirely mistaken
her vocation.
QUEEN'S NURSES FOR SHEFFIELD.
A conference of representative citizens of
Sheffield has been held at the Town Hall, in order to
consider the question of establishing a local branch
of the Queen Victoria's Jubilee Institute for Nurses,
and the influential people who attended, after hearing
an address from Miss Amy Hughes, were so strongly
in favour of the proposed action that the meeting
resolved itself into a committee to take the necessary
preliminary steps. The need of an increase in the
number of nurses for work among the sick poor in
Sheffield, which has long been felt, is thus about to
be satisfactorily met.
nursing at whitstable.
The second annual meeting of the Whitstable
District Nursing Association was held last month.
The association was started with an anonymous
donation of ?.30 for free nursing of the sick poor.
1* urther subscriptions were at once obtained, and a
nurse was engaged. During the first year the
income amounted to ?92 0s. lid., and at the close
of the year the balance in hand was ?19 8s. Id. The
committee were fortunate in obtaining the services
of a first-rate nurse who had for some time been
working in Canterbury, and whose careful attention
to the sick and suffering is much appreciated by all
classes. During 1901 the number of visits made was
3,309 ; during 1902 the number of visits was 3,6G2
and 125 cases were attended. The income for 1902
amounted to ?89 15s. 4d., which included many volun-
tary donations from the poor, and 6ome amounts
from a few paying patients. The committee consists
of the incumbents of the two parishes, and the
Nonconformist ministers of the town with all the
medical men and three ladies.
QUEEN'S NURSES AT SOUTHAMPTON.
The staff of the Queen's Jubilee Nurses' Insti-
tute at Southampton was so pressed with work last
year that the superintendent was obliged to take a
district herself. Six nurses were therefore engaged
in attending the sick poor of the town, and alto-
gether upwards of 12,000 visits were paid. Unfor-
tunately, although in consequence of the munificence
of Mr. Andrew Barlow, the staff was enlarged, the
list of subscriptions for "the twelve months showed a
serious decline instead of the steady rise so urgently
needed. Southampton cannot afford to have its
reputation for liberality in good causes suffer, and
we hope next year to learn that there is a substan-
tial increase in the regular contributions. The
committee are of opinion that the principal reason
why the financial support is inadequate is that the
nature of the work is not sufficiently published
abroad. This should be easy to remedy. Mean-
while, an appeal for a bicycle for the nurses to help
them in getting about more quickly over the large
area which has to be covered, is not likely to fall on
deaf ears.
FOOTBALL AND NURSING.
Redruth and Camborn9 football players have
been helping nursing interests by a football match
played at Camborne, the proceeds of which are
stated to be over ?30. This sum will be equally
divided between the Camborne and Redruth Nursing
Associations.
DRESSED AS NURSES.
Last week at Winchester a woman attired in a
nurse's uniform, was sentenced to three months'
imprisonment for obtaining money by false pre-
tences and hawking without a license. It was stated
that she got a number of girls, whom she dressed as
nurses, to sell books on the pretence that the pro-
ceeds were in aid of a local charity. All this is very
deplorable, and most of all because it shows the
ease with which the careless public can be gulled-
There is no difficulty in ascertaining whether girls
dressed as nurses, posing as the collectors for a
charity, are entitled to the uniform they wear. We
cannot, without legislation, which, if obtained would
be difficult to enforce, prevent unauthorised persons
from masquerading as nurses ; but when they attempt
to make use of uniform for swindling purposes, it
only needs inquiries to defeat their nefarious
purpose.
SHORT ITEMS.
The King has bestowed the decoration of the
Royal Red Cross upon Lady Furley and Mrs. Mary E-
Bruce in recognition of their services in tending the
sick and wounded during the recent war in South
Africa.?The death, in a railway accident at Bar"
berton, in the Eastern Transvaal, is announced
Miss Marion A. S. F. Patteson, of the Army
Nursing Service Reserve. Miss Patteson was the
daughter of the late Rev. T. J. Patteson, of Kin-
nettles, Forfarshire.?The directors of International
Plasmon, Limited, publish a picture advertisement
showing in a striking manner the contrast between
the Mrs. Gamp of the past and the nurse of
present day, which they offer to send to any nurses
applying for it to GGa Farringdon Street, London?
March 14, 1903. THE HOSPITAL. Nursing Section. 327
IE be nursing ?utlooft.
" Prom magnanimity, all fear above;
From nobler recompense, above applause,
Which owes to man's short outlook all its charm."
THE MONTHLY NUKSE.
The statement that one of the lying-in hospitals
trained 127 monthly nurses last year gives an
outlook that fills one with dread for the status of
the profession. For some of these " nurses " receive
six -weeks' training and a certificate for a fee of
seven guineas, and somewhat similar terms exist
at other lying-in hospitals; at Queen Charlotte's
tHe fee is fifteen guineas for twelve weeks, but the
variations are of little account. The great trouble of
to-day is that the term " nurse " is of such general
use that it has no special significance ; it may mean
the little Board-school girl of fourteen just at her
first place to " mind the biby " and paid 2s. a week ;
0r it may mean some nice old fat slum muddler, who
fias her "monthly certificate " hanging in her room
and who " sees her neighbours through " when the
baby arrives for an inclusive fee of 5s.; or it may
mean a stately, refined woman, sitting in her office
and directing some enormous business and con-
trolling some hundreds of human beings ; or it may
mean the highly-skilled nurse, following Sir Frederick
Treves on to the very field of battle. How is the
to discriminate between these nurses ?
^he profession itself has an unformulated law that
^l,ee year3' training in a general hospital is the only
Salification that entitles to the use of the term
drained nurse." This they consider is the basis on
^lich all frrther teaching should rest, and the nurse
stV trained -an then begin to specialise if she likes,
^.nd if oily trained nurses were taken at the lying-
lri hospitals?nurses knowing already the routine
WorA- of tendance on the helpless and of ward work
genera-ijr, there would be no objection to only a six
Peeks' or a three months' training in the special line.
But women are taken into the lying-in hospitals to
train, who have no previous experience whatever,
and what is worse, who have many of them picked
UP the superstitions of the lower classes, and who
pass them on from one to the other. No one has
forked long in a maternity ward without coming
across the elderly sniffy and highly respectable pro-
bationer, who assures everyone that she knows all
about babies, and is only there because doctors
for their own reasons, now insist on trained
nurses. "When the sister is not looking this " ex-
perienced " woman will show some young proba-
tioner how to " squeeze the milk " out of a baby's
breasts, or will state with regard to a non-thriving
child that of course it wont live because it was not
given castor oil directly it was born. The training
needed to guard a nurse against the horrible super-
stitions that surround the labour room should be long
and thorough, and should be founded on theory as
well as on experience. What theoretical knowledge
of physiology or hygiene have those 127 nurses
turned out of the hospital last year 1 And the
experience of hygiene in a hospital is not readily
adaptable to hygiene in a private house. The
nurse may find dustless wards, daily clean linen, ever-
open windows in hospital, and accept them as part
of the ordinary routine, and yet have no theory of
the danger of dirt and stuffiness on which to base
her manner of keeping a lying-in room at a private
case. We know of one case in which a dim idea of
the value of cleanliness had been instilled into a
monthly nurse, but so faulty was the teaching that
on arriving on the scene just after the birth of the
baby, she proceeded to sweep the room while waiting
for the medical man to come and express the pla-
centa ! Again, these monthly nurses are sent to
cases in the country, and there complications may
arise with which they are quite unfitted to deal; for
instance, it may be necessary to give the mother
chloroform while the doctor uses the forceps; this is an
easy matter for the trained nurse under the direction
of the doctor, but it is a terrible position with only
a six weeks' trained woman to help. There is no
need to multiply these stories ; it is impossible to go
about much and not meet with them constantly ;
they can only be stopped by the better organisation
of the nursing profession. At present there is no
body of nurses here, as there is in America, which
can definitely say this or that thing shall, or
shall not be in the nursing world. The young nurse
fresh from her training, and pleased with herself and
her hospital, does not take a wide enough outlook
to see how her future is jeopardised by the want of
coherence amongst nurses as a whole. The elderly
nurse looks on at the jealousies and petty spites ; the
effort of this woman to thrust herself forward and
advertise herself no matter how the nursing cause
may suffer; or the indolence of that woman, who,
having attained a matron's position, retires into an
idle and selfish existence, and the elderly nurse
grows hopeless and sad. We have spoken before of
the need of loyalty in a hospital; but there is an even
greater loyalty demanded of nurses, and that is to be
true to their vocation as a whole, to work for the
nursing profession as self-lessly and as truly as they
work for their patients. Until all petty aims and
angers are buried, until personal feelings are made
subservient to professional ones, we shall never get a
body of women strong enough to govern themselves
and to protect their calling. When we do get that
body?and may it come quickly !?we shall be able
to stay this anomaly of the six-weeks' trained nurse ;
we shall be able to regulate the period of training
and perhaps be able to do away with much of the
ignorance and incompetence that is such a curse at
present to woman in her greatest hour of trial.
328 Nursing Section. THE HOSPITAL. March 14, 1903.
lectures on fIDebical anl> Surgical IRurslng.
By John Hopkins, F.R.C.S., Medical Superintendent of the Central London Sick Asylum District Asylums,
Cleveland Street, W., and Hendon, N.W.
LECTURE III.
Now that you are familiar with the nature of microbes
and with the effect they produce on the body, you are in a
position to study the means whereby their injurious effects
may be prevented.
Long before microbes were thought of our prehistoric
ancestors probably had discovered empirically how to deal
with them.
The rules that prevailed in the dairy were to exclude dust
and wind and all unnecessary movement that will stir it up.
Hence the scrupulous washing of the place, the scalding of
the pan-mugs, the exclusion of all but the dairymaid, the
quietude of her movements, her closing of the ^door behind
her as she went in to shut out all animals, the scalding of
the skimming dish, of her hands, of the butter pats and
wooden scales, and the placing of the butter in fresh cold
water. The use of scorched rag to the cut cord of the new-
born child is another example. The making of jams, potted
meats, pies, and pickles were all in their time great dis-
coveries in the art of preventing food from going bad.
Heat, then, dry or wet, was the household remedy for the
destruction of microbes, and scrupulous cleanliness the means
adopted for their removal. The scientific studies of the past
30 years have confirmed all this, and have added a list of
chemical substances that may be used with eqoal efficacy
where heat would be impracticable. Let these well-estab-
lished rules, then, be applied by the nurse in her calling.
By scrupulous cleanliness let all microbes be removed away,
by heat let them be destroyed, avoid all dust making in the
neighbourhood of the sick, and keep your patients' food
where the domestic animals cannot contaminate it.
Microbes are to be found everywhere ; upon the skin and
mucous membranes. When a wound is inflicted they are
conveyed into the wound, and if allowed to remain there,
they set up inflammation in it and a free flow of pus from it.
When they have thus got a hold upon the cut surface they
are not so readily got rid of, and instead of the wound healing
as you generally see it do after a surgical operation, straight
away, it closes by growing up from the bottom by a long
and tedious process that takes weeks instead of days to
accomplish. A wound that heals up from the bottom does
so by granulation; that is, the cut surfaces present little
heads all over it, about the size of large pin-heads, or small
grains of sago, which grow one on the top of others till the
wound is filled and closed.
In order to get a fresh-made wound to heal at once by the
raw surfaces adhering to one another, microbes must be kept
out of it or, if they are already in it when the wound comes
nnder observation, they must be got out of it as quickly as
possible. A wound inflicted by any means, except when
under antiseptic precautions, will contain them, and they
can only be igot rid of by free washing with an antiseptic
solution, that is, a fluid that will destroy what microbes it
fails to wash away. The most powerful solution and the
quickest is that of perchloride of mercury, but care must be
taken in the use of it as it is very poisonous and is readily
absorbed. Coal-tar preparations are good, being both power-
ful and comparatively harmless; but carbolic acid may be
absorbed and cause a mild poisoning, the evidence of which
is found in a smoky discoloration of the urine.
When a wound has to be made by a surgeon, the part to
be operated upon needs washing with soap and water, the
soap being necessary to remove the natural fat of the skin
that is secreted by the sebaceous glands. This may be
supplemented by ether, which is a powerful fat solvent.
When the skin is washed it should also be scrubbed with a
brush to remove the loose outer scales of epidermis. It
should be bathed also with an antiseptic solution, and then
covered with a dressing moistened with an antiseptic and
kept covered with it for a few hours before operation. In
order to be quite free of epidermal scales when the patient
is brought to the operation table after a part has been
covered for two or three hours in the manner just stated, it
is well to wash it once more, for by that time the outer
epidermis has softened and may be washed off in abundance.
It is necessary to deal in the above manner, not only with
the part actually to be incised, but with all the skin that is
to be handled by the surgeon or that may come in contact
with the instruments that are to be used. At the operation
itself, diseased parts, where possible, such as a gangrenous
foot, must be already enclosed in absorbents, and these
again in impervious material, such as mackintosh cloth, so
that there may be no fear of contamination; and the part
to be exposed must be surrounded with linen recently boiled
and rendered antiseptic, [inside of which clean mackin-
toshes should be folded. Thus prepared, the danger of
microbes being conveyed from the surrounding skin
or clothing of the patient into the wound is pre-
vented. Given a wound free from microbes, care has to
be taken to prevent their entry during the process of
healing. For this purpose, dressings must be applied over
the wound. What these are and how they should be cut,
prepared and put on you will learn elsewhere. At the
present moment it is sufficient to indicate the principles that
should guide you. The dressings must be soft, t^enly
applied, and sufficient to cover the wound, and a p0od area
beypnd; absorbent, that they may'soak up the discharge,
thick enough to absorb all of it without becoming sodden-6^*
They should be applied firmly enough to exclude the adna^"
sion of air, but not to constrict the bandaged1 part, and :^e
frequency with which they should be changed depends uj??n
the quantity of discharge. Generally after an oj.-.ration thlat
has been aseptic, they need only be changed when tr^e
stitches have to be removed. (
When a nurse has the care of instruments, sutireg> and
ligatures, sterilisation of all by recent boiling bif0.i-e use,
and again after use, before the blood, etc., has become hard
upon them, is necessary. Transfer them from the steriliser
directly one by one to the lotion in which they must lie well
covered by it, ready for use. There is one warning word as
to knives, scissors, and needles : too much boiling and anti-
septics blunt them. So wash and scald these well and keep
covered in an aseptic cloth.
Antiseptics for mucous membranes need no further men-
tion here, than that their application to the throat needs
practice to acquire the requisite skill to do it effectually>
and that their use is often attended with the happiest results
when properly applied.
It should be remembered that if a wound becomes infected
antiseptics are still necessary, for however many kinds
microbes there may be in a wound, there are yet other kinds
to be excluded.
Iimants ani? TKflorfterg.
Lounge Chair.?Sister Vida, 2G Francis Street; ^'F'l
appeals for a second-hand reclining chair for a young
woman in consumption who is to undergo the Fresh-air Core*
She is quite unable to pay for one herself.
March 14, 1903. THE HOSPITAL. Nursing Section. 329
fIDr. 5\>t>ne? Ibollanb on Morltftouse IHursing.
The 31st annual Central Poor Law Conference was held in
the council chamber of the Guildhall on Tuesday and
v ednesday under the presidency of the Hon. Sydney
Holland.
The President's address dealt exclusively with the report
of the departmental committee on the nursing of the poor in
Workhouses. He said that there was a widespread and deep
eeling throughout the country that the sick poor ought to
ke properly nursed, and he felt very strongly that it was not
<Jnly the duty of the country to insure this, but that it was
ad social economy, to put it on the lowest ground, not to
?cure and get back to his work any breadwinner stricken by
Alness, or a mother of a family; and it was bad economy to
let little children grow up to face life maimed and weakened
y illness. Wherever they had sick people there should be
a Properly trained nurse, and, that being so, they must either
take the nurse to the sick or the sick to the nurse. If they
granted this, any board of guardians who failed to carry it
out should be compelled to do so ; and if the Local Govern-
ment Board had no present power to enforce it they
?should apply to Parliament and get powers, and the
"whole feeling of every right-minded person would back
them up. He had great hopes that Mr. Long would take
^hat was good in the report of the departmental committee,
and would not only be content with merely makiDg recom-
mendations, but would issue an order to guardians to carry
?ut the report. He expressed regret that on the committee
^hich framed the report there were no experts in nursing.
he report contained an excellent suggestion as to the
bringing of the sick to the nurse by the amalgamation of
small infirmaries, and there was the still more feasible plan
^ sending the sick from the small workhouses to the
Nearest cottage or general hospital and paying for the
accommodation. Both these plans, if carried out, would
lessen the difficulty of getting the patients properly nursed
small workhouses. It was a matter of general rejoicing
^hat the committee had recommended that the position of
the superintendent nurse should be better; that she, at any
rate, should be in sole charge of the sick, and no longer
?nder the commands of an untrained matron, and that her
duties should be clearly defined.
The Keduction of Superintendent Nurses.
Eut, to the despair of all, the committee had suggested a
r'ery large reduction in the number of superintendent nurses,
"aQd he earnestly trusted that Mr. Long would resist it. There
Was a trained matron in every cottage hospital in England,
arid many of these hospitals had fewer beds than the small
Workhouse infirmaries. He spoke with some knowledge of
dorses when he said that the main thing which militated
against their getting into the Poor Law service the best of
purses, and which made their position intolerable when in
ak| was the putting of trained women under an untrained
matron. If they wanted to encourage good women to enter
the Poor Law service they must define their duties, improve
their pay, their surroundings, their food, and their con-
ditions of service; and with a view of getting them to stay
the service the Local Government Board should give them
good increases of pay after five, 10, and 15 years' service,
and a pension of full salary after 20 years' service. Twenty
years spent in nursing was a woman's whole life of possible
"Work. The Local Government Board, and not the guardians,
should give the pay and pensions; for he entirely agreed
^ith the recommendation of the committee that a large part
of the expenses of nursing should be treated as the salaries
of officers now were and be paid out of the Exchequer.
The "Qualified Nurse."
Then the committee proposed to abolish the assistant
nurse. It was high time tbat this was done when they
heard of assistant nurses not knowing how to make a
poultice or take a temperature. But they had invented a
very objectionable person, to be called a " qualified nurse."
Any young woman was to have this exalted title if she
had spent one year in a minor training school, which,
t3 put it bluntly, meant a place where she could not
possibly be trained?namely, the sick wards of a small
country workhouse where a superintendent nurse was
employed, and where a non-resident medical officer under-
took to give some of his time to instruct her. They all
knew quite well that at such a place there was neither
a sufficient variety of cases, nor enough of surgical
treatment, nor was there a supply of appliances to give any
training worth the name. A nurse beginning her nursing
life in this way was not at all likely to learn that high tone,
that standard of professional duty and manners towards
patients, which it was of first importance that every nurse
should have, and which could only be acquired at a proper
training school under a properly trained head. It was a
bed-rock principle that it was absolutely wrong to pretend
to train young women where no proper training could be
given, and, that being so at the smaller country work-
houses, it was a fraud on the public to turn out women
from them with the official stamp of qualified nurse on
them, and it was utterly wrong and unworthy of the
Local Government Board to try to get over their short-
ness of applicants by inducing young women to join
for the purpose of getting this title of " Qualified Nurse."
The right way to attract women into the Poor Law service
was to improve the conditions of that service and not attract
women by the glitter of a title which would enable them to
deceive an ignorant public. It was not only wrong to the
public, but it was a gross injustice to the whole nursing pro-
fession who had given three or four years of their lives to
acquire the status of a really qualified nurse to flood the
country with a spurious article "hall marked" genuine.
This was not merely his opinion; it was the opinion of
every matron of every voluntary hospital, and of the matrons
or superintendent nurses of all the big Poor Law infirmaries;
it was the opinion of all the best authorities on nursing in
England, and of many boards of guardians; and he
imagined that no petition had ever gone to any Government
department so influentially signed as the one against this
plan of giving the title of " Qualified Nurse " to unqualified
women.
The Need of a Nursing Department.
He really saw no difficulty in the way of getting nurses
for the small workhouses, if the Local Government Board
would have the pluck to grapple with the subject. It would
be quite feasible to train a very much larger number of pro-
bationers than was done at present at the big Poor Law
training schools, and a number of others could be trained
at the voluntary hospitals. The Exchequer should pay
both the Poor Law and voluntary hospital training schools
for training these probationers. Probationers would sign
an agreement to serve the Local Government Board
for four years. Two to three years of that time should
be spent in the big training school and two periods
of six months at one or at two of the small country
workhouses. The very great advantage of this system
would be that the nurses who went to the small work-
houses would not go there at the commencement of their
330 Nursing Section. THE HOSPITAL. March 14, 1903.
careers, bat after they had had considerable training, and
had learned the tone of a good hospital and the proper
way of behaving to, and treating, patients. This involved
the setting up of a nursing department, and was the only and
inevitable solution of all nursing troubles. If this ever came
about, he prayed that there might be some trained women on
the nursing board and some women inspectors appointed.
The members of the committee seemed to have feared that, if
a nursing department were set up, the Local Government
Board might have a lot of nurses on their hands unemployed.
He felt very sure that employment would easily be found for
all their nurses, even if they had to be sent to relieve some
of the poor nurses who were worn out by looking after too
many patients or who were worried into despair by imbeciles
or epileptics.
EvergDoDp's ?pinion.
[Correspondence on all subjects is invited, but we cannot in any
way be responsible for the opinions expressed by our corre-
spondents. No communication can be entertained if the name
and address of the correspondent are not given as a guarantee
of good faith, but not necessarily for publication. All corre-
spondents should write on one side of the paper only.]
NURSING BY ORDERLIES.
" Reserve Sister " writes: I have no wish to prolong the
discussion on the above subject, but as the writer of the
article which gave rise to it, I cannot allow the letter signed
"Late A.N.S." to pass without a protest against the most
unwarrantable charge of " mud slinging " it contains. The
article in question was a perfectly fair and reasonable
comment on a matter of public interest, dealing with some
of the defects of the existing system (many of which the new
regulations will remedy). It was in no sense an attack on
the orderlies themselves, nor did I imagine anyone would
take it in that light. I have several times said that the
majority of those I bad the pleasure of working with were
painstaking, courteous, and hardworking men. Even had
the reverse been the case, I should have thought that a lady
who had been in the Army Nursing Service, would have been
the first to realise that a sister's position, if nothing else,
would have prevented her from descending into the arena
and indulging in what she rightly terms " the undignified
proceeding " of a mud-slinging contest on equal terms with
an orderly.
A MISLEADING ADVERTISEMENT.
" Justice " writes: I would like to have the opinion of
others in the following case. Lately I was a selected
candidate for the post of " lady superintendent nurse" in
a Union Infirmary. To rightly understand the case it is
necessary to quote two phrases from the advertisement:?
" Age not to exceed 40 years," and " having had at least
five years experience as a nurse in a general hospital." In
applying for the post I mentioned that I was 40, and that
my nursing experience had been gained in general hospitals
till I came to my present post as superintendent nurse in a
Poor Law Infirmary. I was twice interviewed by the
Infirmary Committee, who unanimously recommended my
appointment to the board. The only difficulty they had
was regarding the age, as I was 40 last May, which I did
not hesitate to tell them. After a heated discussion the
board appointed a lady who had received her training and
experience in Poor Law Infirmaries. I did not know this
at the time of the appointment, or I would have drawn the
attention of the guardians to it, as it was distinctly depart-
ing from the second clause above quoted. The decision is
in my opinion neither legal nor logical, for by no stretch of
imagination can a Poor Law Infirmary and a general hospital
come under the same heading. I was led to understand
that the guardians were particularly anxious to have a
superintendent who had been trained in a general hospital,
but I fear that many of them do not know the difference.
appointments.
[No charge is made for announcements nnder this Head, and we are
always glad to receive, and publish, appointments. But it U
essential that in all cases the school of training should be
given.]
Beaminster Union.?Miss Laura Ridsdale and Miss Jane
Rogers have been appointed assistant nurses. Miss Ridsdale
was trained by the Meath Workhouse Nursing Association,
at the Crumpsall Infirmary, Manchester; and Miss Rogers
by the same Association at St. Peter's Home, Kilburn.
Bolton Borough Fever Hospital.?Miss Margaret
Lewis has been appointed staff nurse. She was trained at
the Chester Isolation Hospital, and has since held an
appointment at the Samaritan Hospital for Women,
Nottingham. Miss Derlin Isabella Harley has been ap-
pointed ambulance nurse. She was trained at the Blawart-
hill Fever Hospital, Yoker, near Glasgow.
Brighton Workhouse Infirmary.?Miss Mary Rowell
has been appointed charge nurse. She was trained at the
General Infirmary, Worcester, and the Royal Maternity
Hospital, Edinburgh. She has since had temporary charge
of the infirmary at the Magdalen Hospital, Streatham, and
has done private and district nursing.
Budleigh Salterton Cottage Hospital.?Miss Lewis
has been appointed matron. She was trained, at Bristol
General Hospital, where she was afterwards sister. For the
past nine months she was superintendent nurse of j^the
Clifton District Nursing Association.
Burton-on-Trent Bororgh Fever Hospital.?Miss
Edith Burtenshaw has been appointed matron. She "was
trained at Paddington Infirmary, and afterwards became
sister. She has since been matron of the Western General
Dispensary and of the Colchester Diphtheria Hospital.
Bury (Lancashire) Union Workhouse ?Miss E." A-
Thompson has been appointed staff nurse. She was trained
at Prestwich Union Workhouse Infirmary.
Cheltenham Union.?Miss Ellen Sheen has been ap*
pointed assistant nurse. She was trained by the Meath
Workhouse Nursing Association at Moseley Hall Convalescent
Hospital, Birmingham.
City op London Hospital foii Diseases of the Chest.
Victoria Park, E.?Miss Clara E. Trafford has bee?
appointed matron. She was trained at the North Stafford-
shire General Infirmary and Eye Hospital, where she was
afterwards sister and temporary night superintendent. Sbe
then took up private nursing as a member of the Registered
Nurses' Society, and has since been assistant matron at tbe
City of London Hospital for Diseases of the Chest.
Leeds Union Infirmary.?Miss Elizabeth Thorn ha&
been appointed charge nurse. She was trained at Barnl^
Union Infirmary for three years, and has since been cbarge
nurse in the same institution.
Middlesbrough Union Children's Hospital.-?^5
Emma Worthy has been appointed charge nurse. She
trained at the London Hospital, Whitechapel, and
afterwards staff nurse. She has since been nurse at a
private hospital in Manchester.
Rochdale Infirmary.?Miss Sara Ainsworth has
appointed sister. Sho was trained at the Royal Alber
Edward, Wigan, and has since been night sister at Soutbp?r
Infirmary, and sister at the Newport and Monmoutbsbir
Hospital.
March 14, 1903. THE HOSPITAL. Nursing Section. 331
Sefton Park Nurses' Home, Hargreaves Road,
Liverpool.?Miss Golding has been appointed lady super-
intendent. She was trained at the Liverpool Royal In-
firmary. She has since been sister at the Royal Hospital for
Sick Women and Children, Bristol, and night superintendent
and home sister at the Brompton Consumption Hospital,
London.
Sheffield Union Infirmary.?Miss Gertrude Mary
Nash and Miss Caroline Barnes have been appointed sisters,
^liss Nash was trained at Poplar and Stepney Sick Asylum,
and afterwards held the position of staff nurse. Miss Barnes
"as trained at the Union Infirmary, Epsom, and has sines
bsen nurse of the City Hospital, Park Hill, Liverpool.
St. Olave's Union Infirmary, Rotherhithe.?Miss
Ethel Florence Hunter has been appointed charge nurse.
She was trained at Sheffield Union Infirmary, and has since
been charge nurse at Keighley Union Infirmary.
Victoria Hospital, Blackpool.?Miss E. Lewis has
been appointed sister of men's wards and theatre. She was
trained at the Royal Infirmary, Liverpool, and has since been
sister at Corbett Hospital, Stourbridge. She has also done
private nursing.
(Presentations.
Liverpool Hospital for Women.?Miss J. R. Wallace,
night superintendent of the Hospital for Women, Shaw
Street, Liverpool, was on the 6th of this month presented
"ith an occasional chair from the lady superintendent, and a
handsome silver-mounted bread board, with knife and fork
to match, by the sisters and nurses, on the occasion of her
resignation. Miss Wallace was trained at Brownlow Hill
Infirmary, and afterwards became night superintendent of
the Hospital for Women, and although her stay there was
under a year, she was much loved and respected. She is
shortly to be married to the Rev. J. H. Eastwood, B.A., of
forest Hall, Newcastle-on-Tyne.
TRAVEL NOTES AND QUERIES.
Swiss Holidays (S. C.).?Please use a pseudonym when
Writing as well as name and address. As a rule, personally-
conducted parties do not leave for Switzerland as early as you
"!sh ; but watch this column and I will tell you of the earliest
arrangements. The trip j'ou ask about has nothing to do with us^
and I am afraid the gentleman who is organising it will not have
Toom for all those who are applying. However, I have sent you
his address by post. Write and ask him. I know he prefers to
take workers with him rather than drones. So if he has room you
"ill stand a good chance.
A Kind Appreciation (Sister Binnie).?Thank you so much
for your kind letter. It is a reward for many a long hour's work
to hear that I have given pleasure and help to those whose time is
fully occupied, and who need a holiday so much, albeit their purses
are slender. To give a little friendly help to those lightens work
ihat would otherwise be both wearing and dull.
Italian Trip or Switzerland (Irene). ? You are not
troubling me at all, for I like to help those who need it. I
think you must write to me again for your letter puzzles me
rather. You seem to think that a personally-conducted trip is
?going to the Channel Islands, but that is not so, and I do not
? consider those Islands remarkable for cheapness. The Italian and
Swiss trips have nothing to do with us, but if you will tend me a
stamped and addressed envelope I will give you the notices and
vou must write for particulars. Personally, I should prefer the
Italian one, it is not so big, and I am afraid you will be too late
"for the other. Write to me again if I can do anything further
for you.
j?aet Xonbon murses: Hnnual
faceting.
Owing to the steady downpour of rain on Tuesday after-
noon the attendance at theiMansion House in support of the
East London Nursing Society was very small; indeed, the
nurses themselves outnumbered the non-uniformed visitors.
The Lord Mayor, accompanied by the Lady Mayoress, pre-
sided, and there were on^the platform the Bishop of Barking,
Sir Dyce Duckworth, M.D., LL.D., the Rev. Dr. Hermann
Adler, the Mayor of Stepney, and others. The Lord Mayor
having briefly urged thejclaims of the society, the Bishop of
Barking proposed " that the annual report and audited
balance-sheet as now submitted be adopted, and that the
general and executive committee be re-elected."
The work of the Society, the Bishop said, needed no
advocacy where it was known ; the misfortune was that it
was known so little. Speaking from personal experience,
he bore testimony|to thejvalue of the work, not only among
" bedridden folk " and those who had undergone operations,
but among patients whom it was impossible or undesirable
to admit to the hospital at all. The nurses were no experi-
mentalists, but fully trained: they covered an enormous and
growing area, and he earnestly pleaded for additional funds
to enable them to carry on the work.
Sir Dyce Duckworth said it was some ten years since he
had taken part in| the annual meeting of the Society; he
had, however, never'ceased to take an interest in it; and as
one of the Council of Queen Victoria's Jubilee Institute, he
had opportunities of watching its progress and development.
There were certain conditions to be fulfilled before any
society could be affiliated to the Institute, and he was glad
to be able to say that they were fulfilled by the East London
Nursing Society; it was a feather in the cap of a society to
have the segis of the Institute over it. It was the duty of the
Institute to criticise as well as commend; and although
Rome was not built in a day, the members of the Council
thought the Society was now capable of improvement
in two directions. They both depended upon funds,
and the highest wishes of the Society itself would
be fulfilled when they were carried out. The nurses ought
to be better paid, and they ought to have a home. As
matters now stood in the nursing world, these nurses were
competing with those who could secure better pay. It was
a matter which could be easily rectified by increased con-
tributions from the public, and those responsible for the
Society's working would be only too happy to increase the
scale of pay. One disadvantage was that the salaries did
not enable the nurses to contribute anything worth mention-
ing to the Royal National Pension Fund, or to make any
provision for the dark days of the future. The other need
was a central home. It might be good for the patients to
have the nurses in scattered houses, but it was bad for the
nurses; they needed comforts of good cooking such as it
was not easy to get in squalid lodgings or small houses.
"When a nurse comes in on a day like this," said Sir
Dyce, " she meets with a very poor return for all the out-
pourings of her heart during the day if she has to return to
a dreary lodging."
The Chief Rabbi proposed the following resolution, which
was seconded by the Mayor of Stepney:?
" That this meeting acknowledges the value of the work
done by the 'East London Nursing Society,' and undertakes
to make its claims more generally known."
The Lord Mayor said he sympathised with the aims of the
Society to the extent of ten guineas, and he had no doubt
the Sheriffs felt equal to five guineas each. A vote of thanks
to the Lord Mayor and Lady Mayoress was proposed by the
Rector of St. George in the East and carried unanimously,
and the meeting terminated.
332 Nursing Section. THE HOSPITAL. March 14, 1903.
Echoes from tbe ?utsi&c Morlii.
Movements of Royalty.
On Monday the King held a Levee at Buckingham Palace
in the morning, when the first presentation was that of
Captain H. C. French, R.A.M.C., to whom the King gave the
decoration of the Albert Medal, Second Class, for bravery in
jumping overboard, in the Straits of Malacca, after a native
fireman. Captain French, at the time, was a passenger on
board the transport Wakool, and the fireman was saved. In
the afternoon the Queen, Princess Victoria, Prince and
Princess Charles of Denmark, and the children of the Prince
and Princess of Wales, arrived from Sandringham. The
Queen drove from St. Pancras with Princess Victoria of
Wales on her lap, and Prince Edward also accompanied his
grandmother. In the next carriage were Prince and Princess
Charles of Denmark, with their two nephews, Princes Albert
and Henry, and special interest was taken in the third
carriage, which contained Prince George of Wales, who
thus made his entrance into London. Tuesday was the
anniversary of the wedding day of the King and Queen, and
though ifc is almost impossible to believe it, so youthful
does the Queen still appear, the marriage took place 40 years
ago. There was a large family dinner party at Buckingham
Palace in the evening, and a small dance, to which about
400 invitations were issued, followed. Pleasant festivities
took place at Sandringham and Windsor.
The Ameer and His Subjects.
The Ameer has been making some sweeping reformations
in Afghanistan. He has proclaimed by beat of drum that
no one of his subjects shall for the future have more than
four wives, and that all wives in excess of that number shall
be divorced. The proclamation adds that any breach of the
Mohammedan Law in this respect shall be severely dealt
with. The Ameer has set an example himself by divorcing
all his wives except the regulation four. He has allowed
any of those divorced to remarry at their pleasure, and has
also informed them that those who do not marry will
receive sufficient to support them for life. To induce
religious tolerance he sentenced a man who was brought
before him charged with taunting another meat-seller on
account of his religion to be blown from a cannon, and has
proclaimed that a like punishment awaits all who commit a
similar offence. To put down robbery he has condemned
four robbers who have been captured to be placed in iron
cages and hung up in several prominent thoroughfares as a
warning to offenders. To prevent the undue accumulation
of wealth or miserliness he has proclaimed that all who
possess more grain than they require to keep their family for
four months shall sell it. If not, the whole amount will
be taken by force. The Ameer has increased and varied
the food of the Sepoys, and thus greatly enhanced his
popularity with this particular section of his subjects.
Mr. Balfour and the Bible Society.
Not since 1815 has an English Prime Minister advocated
the claims of the British and Foreign Bible Society until
Mr. Balfour spoke at the inaugural meeting in connection
with the centenary at the Mansion House on Friday. Another
remarkable feature of the gathering was the presence of the
Lord Mayor, who professes the Hebrew faith, in the chair.
The Prime Minister dwelt on the extraordinary work done
by the Society and the great benefits resulting therefrom to
philology and the allied sciences. He went on to say that
religion was its real force, and contended that an ever-in-
creasing knowledge of the other great religions of the world
and of the history of the period immediately subsequent to
the beginning of the Christian era, so far from rendering
the Bible less interesting or valuable to us from a religious
point of view, greatly augmented in every respect the value
which it must have for an educated community. These
researches, he affirmed, made it far more of a living record
of the revelation of God to mankind than it ever was or
could be to those who, from the nature of the case, had no
adequate conception of the circumstances under which that
revelation occurred or of the peoples to whom it was
vouchsafed.
The Author- of " John Inglesant."
The death of Mr. Joseph Henry Shorthouse took place last
week. The author of "John Inglesant" had been out of
health for a long time, but it was not known by his many
admirers that he was seriously ill. Mr. Shorthouse wrote
several books, but " John Inglesant" is his lasting title to
literary celebrity. This brilliant and remarkable work took
him 20 years to write; that is to say, when he was not
immersed in his business as a manufacturer of sulphuric
acid at Birmingham, he spent his leisure in weaving the
romance which, though only intended for private circula-
tion, took the public by storm at the time it was published
by Mr. Macmillan, and reached in a few years a circulation
of between twenty and thirty thousand copies. Mr. Shorthouse
was a shy, retiring man, with an impediment in his speech,
who seldom went into society, but was devoted to his wife,
his home, and his own beautiful dreams. Withal he never
neglected his business, and his goodness of heart was mani-
fested by constant acts of kindness. The funeral was on
Tuesday.
A New Commissioner of Police.
Simultaneously with the retirement of Sir Edward
Bradford from the post of Commissioner of Police of the
Metropolis, it was announced that his successor had been
appointed in the person of Mr. Edward Richard Henry.
The retired Commissioner discharged his very onerous duties
in the most admirable manner for twelve years, and his
well-known figure, with one armless sleeve?he is a soldier,
but he lost his left arm in a conflict with a tiger?will be
much missed. Mr. Henry is a civilian, who has been
Assistant Commissioner for less than three years, and came
to Scotland Yard fresh from India, where, however, he
served with distinction for eight years as Inspector-General
of Police in Bengal. He is in the prime of life, having been
bom 53 years ago, and his great experience in the detection
of criminals by the finger-print system is only one of his
many qualifications for an office in which great sagacity,
patience, and firmness are required. The Commissioner
has command of about sixteen thousand men.
Mr. R. W. Macbeth, R.A.
The new Royal Academician in place of the late Mr.
H. T. Wells is Mr. Robert Walker Macbeth, who was born
in Edinburgh in 1848, and at the age of 23 came to London
in order to join the staff of the then newly established
Graphic. Mr. Macbeth first exhibited at Burlington House
in 1874, and he has not missed a single year since that time.
At the Paris Exhibition in 1889 he was awarded a gold
medal for his etchings and a bronze medal for his painting-
In 1890 his picture, " The Cast Shoe," which now hangs in
the Tate Gallery, was purchased by the Chantrey Trustees
for ?G30. He was elected an Associate in 1883, in which
year he exhibited " The Sacrifice," a girl parting with her
long hair to an old-fashioned wig-maker. His other most
popular pictures are "The Cambridgeshire Ferry," "Cider
Making," " Sheep Shearing," " Landing Sardines," and
" Sparklets," which was quite one of the favourites of the
Academy Exhibition in 1897.
March 14, 1903. THE HOSPITAL. Nursing Section. 333
]for IRcabing to tbc Sicfc.
THE DISCIPLE AND HIS MASTER.
Thou gavest me no kiss,
Jesus, my Master, oft I sadly thought!
Perchance Thou choosest to be found unsought
And I was ever seeking ! Yet in this
Methought, I cannot change; and should I miss
Thee on Thy way, yet there will I abide
And track Thy footprints to the dark stream's side.
Thou gavest unto me
No sign I I knew no loving secret told
As of to men beloved, and I must hold
My peace when these would speak of converse high;
Yet would I, Jesus Master, still be nigh
When these would speak and in the words rejoice
Of them who listen to the Bridegroom's voice.
I never heard Thee say,
" Bring forth the Robe for this My Son, the best I"
Thou gavest not to me as unto guest
Approved, a festal Mantle rich and gay;
Still singing, ever singing, in the cold
Thou leavest me without Thy Door to stay,
And the night draweth on, the Day is old,
And Thou hast never said, " Come in, My Friend: "
Yet once, yea! twice, methinks, Thy Love did send
A secret Message, " Blessed unto the end
Are they that love and they that still endure: "
Jesus, my Saviour, take to Thee Thy poor,
Take home Thy humble Friend !?D. Qrecnwcll.
Let me meet Thee at the door of life, that Thou mayest
be my interpreter through all the way. When crosses lie
before me, and I call them accidents, interpret Thou to me;
show me that the Cross is the road to the Crown. When
weakness overtakes me and I call it failure, interpret Thou
to me; show me that Thy strength is made perfect in
weakness.? George Nathcson.
If we have made our peace with God, if no sin rests upon
?ur consciences which clouds over or hides God's lovirig face
from us, then we may simply accept a natural fear or dread
?f death as a pari of the discipline God knows to be needful
tor us. We have a sure and steadfast promise from Him
who cannot err?the promise of His presence with us.
" Though I walk through the valley of the shadow of
death, I will fear no evil, for Thou art with me."?Bishop
Wilkinson. ? >
Be sure that the heavenly life will be no listless, useless,
vacant contemplation of God ; not mere freedom from pain
and sin, but some active employment, some blessed work
will be ours. Work is a necessity of active life, toil is the
result of the Fall. What that employment will be, in this
life we shall never know; but we doubt not each faculty
will be employed; whilst never through the infinite ages
shall man reach the limit of a work whose freshness and
beauty and interest eannot fail.?T. B. Dover.
flotes anb Queries.
The Editor Is always willing to answer in this column, with oat
any fee, all reasonable questions, as soon as possible.
Bat ttie following rules must be carefully observed:?
x. Every communication must be accompanied by the name
and address of the writer.
?. The question mast always bear upon narsing, directly it
indirectly.
I! an answer is required by letter a fee of half-a-crown must ba
enclosed with the note containing the inquiry, and we cannot
undertake to forward letters addressed to correspondents making
inquiries. It is therefore requested that our readers will net
?nclose either a stamp or a stamped envelope.
Midwives Act.
(172) 1. I have been reading about the Midwives Act in the
Nursing Section of The Hospital, and. as I am about to work up
for the L.O.S., I should be very much obliged if you will kindly tell
me if that society's certificate will hold good when the Act comes
into force, and if any other registration will then be necessary ?
2 I shall be much obliged if vou will give me the address of the
Health Society.?Southsea.
!? Miss Honnor Morten has just added a chapter to the "Mid-
wives' Pocket Book " on this verv important subject. As the
price is only Is. all interested should obtain a copy. The L.O.S.
certificate will be reckoned in Class I. under the new Act, conse-
quently the holder will be fullv recognised. 2. The address of the
National Health Society is 53 Berners Street. W.
L.O.S. Examination.
(173) 1. Can you tell me of any periodical which publishes the
last questions of the L.O.S. Examination ; 2. and a useful coaching
book ??E. S. S.
1. Write to the Secretaiy of the London Obstetrical Society,
20 Hanover Square, VV. 2. See reph to Southsea.
Dispenser.
(174) I wish to qualify as dispenser in the shortest possible time.
Can you tell me where I could be prepared for the necessary
examinations??M. R. IV.
You can obtain all particulars relating to dispensing by applying
to the Secretary, the Pharmaceutical Society, 17 Bloomsbury
Square, W.C.
Sister and Charge Nurse.
(175) May I ask what is the difference between charge nurse
and sister ??I. S.
A charge nurse has the charge of a ward, whilst a sister super-
vises the charge nurses of perhaps several wards.
Maternity.
(176) I am anxious to train as a monthly nurse. If I get my
certificate from a good lying-in hospital is that sufficient to enable
me to join an institution without any general training ??Mary.
A11 the best nursing associations require general training as
well as the maternity certificate. If you could possibly manage
to take a course of training at any of the special hospitals for
women, as well as the maternity certificate, it would be greatly to
your advantage. The Hospital for Women, Soho Square, for
instance, gives a two years' course and a salary of ?8 for the first
year and ?14 for the second, laundry and uniform included.
Home for Inebriates.
(177) Can you tell me where a home for inebriates in a cheerful
locality may be found for a gentleman, and also where the wife
would be admitted as a paying guest ? M. S.
There will probably be many private homes where such a conple
could be received on the terms desired, but you can only hear of
them through advertisement or through private introductions.
Standard Kurslng Manuals.
" The Nursing Profession : How and Where to Train." 2s. net;
2s. 4d. post free. *
"Nursing: Its Theory and Practice." (Revised Edition). 3s. 6d.
post free.
" Surgical Ward Work and Nursing." (Revised Edition). 3s. 6d.
net.; 3s. 10s. post free.
" Practical Handbook of Midwifery." (New Edition). 6s. net;
6s. 3d. post free.
"Notes on Pharmacy and Dispensing for Nurses." Is. post free.
" Fevers and Infectious Diseases." Is. post free.
" The Art of Massage." (New Edition). 6s. post free.
334 Nursing Section. THE HOSPITAL. March 14, 1903.
Gravel iRotes.
By Our Travelling Correspondent.
CXVIII.? IN THE SHADOW OF THE DUOMO.
Close by the stately pile of the Cathedral, and a little
behind the headquarters of the Brothers of the Miserecordia,
lies a little street still called the Via della Morte, from a
tradition that lingers there connected with the apparent
death and resurrection of the beautiful Ginevra degli Amieri
?a tender story of faithful love stronger than death, which
one meets in many old Italian chronicles, a story told
with slight variations, but the main facts of which are
always the same. It is related with the fullest detail by
Boccacio, though assuredly one hardly expects to find so
touching and beautiful a tale amongst his revolting stories.
Ginevra was beloved by one Rondinelli, who was of inferior
birth; the lady's friends, therefore, would not listen to the
prayers of the lovers, and married her, in the high-handed
manner of the day, to a middle-aged suitor called Francesco
Agolanti. Shortly after Ginevra fell a victim to the fearful
plague which raged with such ivirulence in Florence, and
the wonderful account of which we owe to the same pen.
In the introduction to the " Decamerone," there is such a
description of that time as makes one realise, as no other
writer has done, what an appalling scourge it was, and
how the demoralisation caused by its ravages seemed to
stifle human sympathy, and^even natural love, in the hearts
of the stricken Florentines.
Ginevra, supposed to be dead, was hurriedly buried in the
Agolanti vault in the cemetery of the Duomo, which existed
between the western end of the church and where the
Campanile now stands. In the dead of night consciousness
returned to her, and with desperate efforts she burst the
bonds of her grave clothes (there was probably no coffin),
raised the stone which closed the vault, and fearfully stepped
forth, wending her way to her husband's house in the
Corso degli Adimari. To reach this, she passed along the
street now called the Via della Morte?hence the name.
Arrived at her home, the terrified girl knocked at the door,
but her husband, believing her to be a spectre, remained deaf
to her entreaties. Then she fled with trembling steps to her
parents' house, behind the Mercato Yecchio, where she
received the same treatment. Nor is this surprising. Even
in the present day well-educated Italians have an extra-
ordinary fear of death, and [should you be ill and in any
danger of death the Icircumstance is hidden from friends
and acquaintances lest the house should be shunned. I have
been very ill in Venice, so I speak of what I know from
personal experience. Poor Ginevra, a wanderer and an out-
cast, denied by those to whom she should have been so dear,
exhausted by the illness which had so nearly proved fatal^
and terrified by her position, sat down to die indeed on the
steps of San Bartolommeo. Then the thought of her
chivalrous lover came to her, and once more she started up
in the hops that true love would shelter her when family
affection and the vaunted tenderness of a husband had failed.
Rondinelli never hesitated; love, stronger than the fear of
death, welcomed her with unstinted joy, and in his house, in
the care of his aged mother, Ginevra remained till her
marriage with Rondinelli was annulled on the grounds that
a^woman who had been dead and buried was no longer a
wife. How often I have thought of this tender old story as
I have wandered up and down the delightful street called
Via Calzaiolo, or the " street of the stocking makers," looked
upon the steps of San Bartolommeo, and pictured that
desolate figure.
"There is no story so perfect as the Ginevra tale. The
dreadful loneliness of the great dome as she awoke beneath
it; he vast haunted stillness, with here and there the
whiteness of a moonbeam; these quiet, gloomy streets at
midnight; the black shadows; these yawning archways,
like the gates of tombs; the trembling, hunted, heart-
sick thing, with her bare feet wounded on the stones
and the grave clothes falling from her shivering
limbs; everywhere denial, incredulity, horror, super-
stition; everywhere the closed wicket and the cry of
terror as at some unearthly apparition. Then at last the
lover's threshold, the timid summons of despair, the open
door, the instant welcome ; not a doubt, not a question, not
a fear?what matter whether living or dead, of heaven or of
hell ? What matter whence she came ? What matter what
she brought ? Welcome, thrice welcome, as flowers in the
Maytime I Welcome and precious?since the face was hers !
?Pascarel."
Giotto's Tower.
If there is a fine sunset, go to the Piazza del Duomo and
see the wonderful effect of the reflection on the west front
of the church and on the Campanile; they glow, in the warm
rays, like that delightful old-fashioned gem called the fire
stone. Rose, yellow, mauve and flame-colour rays seem to
scintillate all over the marble and dazzle one with their
superb radiance. The beautiful bell tower was designed by
Giotto, hence it is often called the Shepherds' Tower, but he
did not live to complete the work, which, begun by him
shortly before his death in 1336, was finished by Andrea
Pisano and Francesco Talenti. Ruskin calls it " the model
and mirror of perfect architecture." ..." That bright,
smooth sunny surface of glowing jasper, those spiral shafts
and fairy traceries, so white, so faint, so crystalline, that
their slight shapes are hardly traced in darkness on the
pallor of the eastern sky, that serene height of mountain
alabaster, coloured like a morning cloud, and chased like a
sea shell."
As is common in Italian towers, the number and richness
of the windows increases towards the top. All round the
base of the structure runs a band of most delicate little
bas-reliefs of small dimensions; these were most, if not all,
designed by Giotto himself, but the execution was Pisano's,
with the exception of some on the north side both designed
and executed by Luca della Robbia considerably later.
On the eastern side of the Piazza is a house which has
built into its wall the Sasso di Dante, that is to say the
stone of Dante. On this stone, tradition says, he was wont
to sit on summer evenings and jwatch the work of the
builders on the Duomo.
On March 25th a curious ecclesiastical show goes on?
one can hardly call it a sacred service. It has some
mysterious and occult connection with the exploits of a
crusading member of the powerful Pazzi family and also
with the prospects of a good harvest. The exact proportions
of the relative merits of these conflicting ingredients in
the interest of the spectacle I cannot define, but what
happens is this: a huge crowd, chiefly country people,
assembles in the Piazza to watch the explosion of the Pazzi
car, which is stationed near the Baptistery. During the
celebration of High Mass the Archbishop comes out to bless
the car ; almost immediately a mechanical dove flies along a
wire stretched from the high altar and mysteriously
explodes a numbertof fireworks in the car. If the pyrotechnic
display is satisfactory there will be a good harvest; if not,
" Well, if not there won't," as our waiter lucidly explained
to us. It is always a matter of difficulty to extract an
explanation in Italy?there is a delightful vagueness always
present with their information ; as Howells says, after pro-
longed residence there, " We know nothing with certainty! "

				

